# Synthesising enablers and barriers to hepatocellular carcinoma surveillance—A systematic review of qualitative findings

**DOI:** 10.1371/journal.pone.0313216

**Published:** 2025-01-10

**Authors:** Dina Moussa, Joan Ericka Flores, Joseph S. Doyle, Marno Ryan, Jack Wallace, Jessica Howell

**Affiliations:** 1 University of Melbourne, Australia; 2 St Vincent’s Hospital Melbourne, Australia; 3 Disease Elimination Program, Burnet Institute, Australia; 4 Department of Infectious Diseases, Alfred Health and Monash University, Australia; 5 Australian Research Centre in Sex, Health and Society, Latrobe University, Australia; 6 Centre for Social Research in Health, UNSW Sydney, Australia; Shaanxi Provincial People’s Hospital, CHINA

## Abstract

**Background & aims:**

This systematic literature review of qualitative findings aims to identify the perceived barriers and enablers for hepatocellular carcinoma (HCC) surveillance from patient and clinician perspectives.

**Methods:**

A systematic search of databases using key term combinations with the following inclusion criteria: 1) qualitative and quantitative (survey) studies exploring barriers and enablers of HCC surveillance, and 2) qualitative and quantitative (survey) studies exploring barriers and enablers of enagagement in clinical care for patients with cirrhosis and/or viral hepatitis.

**Results:**

The search returned 445 citations: 371 did not meet the study criteria and were excluded. 74 studies proceeded to full-text review, leading to 21 included studies (15 studies from searching with a further six studies from citation review) progressing to data extraction by two independent reviewers. Results from studies exploring patients’ perspectives reinforce that barriers are experienced by patients across different health settings, cultures, and regions. Logistical barriers including costs and transportation, and knowledge/awareness barriers were commonly identified. Studies that included clinician perspectives highlighted the need for healthcare provider education and system-level interventions to optimize HCC surveillance uptake in clinical practice.

**Conclusion:**

These findings highlight the critical need for interventions that enable engagement in HCC surveillance in health services.

## Introduction

Hepatocellular carcinoma (HCC) has a significant global health burden and high mortality rates [1]. It is currently the fourth leading cause of cancer-related deaths worldwide [2]. Hepatitis B and hepatitis C virus infections are the predominant risk factors for HCC globally [3]. The risk of developing hepatitis B and/or hepatitis C related HCC increases when combined with coinfection with either virus, diabetes mellitus, older age, alcohol use and smoking. Alcohol related liver disease and the epidemic of metabolic associated steatotic liver disease are also key drivers of HCC globally [1, 3]. There is a disproportionate burden of HCC in low resource settings such as Eastern Asia and sub-Saharan Africa, with an estimated 85% of global HCC cases occurring in these regions due to the high prevalence of hepatitis B [1, 2].

Regular HCC surveillance with six monthly liver ultrasound is vital for the early detection of HCC where it is more likely to be curable and is shown to improve survival [4–7]. International guidelines recommend six monthly targeted liver ultrasounds with or without serum alpha-fetoprotein (AFP) for people at an increased risk of HCC development [5–12]. The rationale for HCC surveillance is to identify early-stage HCC (Barcelona Clinic Liver Cancer [BCLC] stage 0-A) in patients with preserved liver function that would be amenable to curative treatments, such as surgical resection or ablative therapies [8, 13]. Very early-stage HCC (BCLC-0) has a reported 5-year survival of 86%; however, HCC diagnosed at stages A, A1, and B has five-year survival rates of 69.0%, 56.9%, and 49.9%, respectively [14].

Despite international guideline recommendations, the uptake of HCC surveillance remains unacceptably low [15–20], with a pooled estimate of 24% of people with cirrhosis in a meta-analysis of 24 studies from North America, Italy, the Netherlands, Switzerland, Taiwan, Spain, the UK and Australia [17] accessing surveillance [1, 2].

To improve HCC surveillance uptake, it is essential to understand the perceived barriers and enablers to HCC surveillance from both patient and clinician perspectives to inform health service interventions. In this systematic review of qualitative data, we aimed to analyse this knowledge gap by describing the perceived and experienced barriers to and enablers of HCC surveillance uptake from both patient and clinician perspectives.

## Materials and methods

The primary outcome of interest was to identify barriers and enablers to HCC surveillance uptake. The study design was a systematic literature review of qualitative studies and qualitative data captured within quantitative survey studies in accordance with the Preferred Reporting Items for Systematic Reviews and Meta-Analyses (PRISMA) guidelines for reporting systematic reviews [21]. The PRISMA checklist was used to report the findings of this review.

### Eligibility criteria

Two investigators (DM and JEF) selected potentially relevant studies for data extraction in accordance with the PRISMA guidelines, set for populations of people living with viral hepatitis and/or cirrhosis. The inclusion criteria were as follows: 1) qualitative and quantitative studies exploring barriers and enablers of HCC surveillance, and 2) qualitative and quantitative studies exploring barriers and enablers of care for patients with cirrhosis and/or viral hepatitis. Studies included surveys, focus groups, and interviews with patients, clinicians, or both. Only quantitative studies that included qualitative or open-ended questions and self-expressed free text written or oral responses in surveys were included, and only data from these responses were included in the study.

### Search strategy

**Fig 1:**
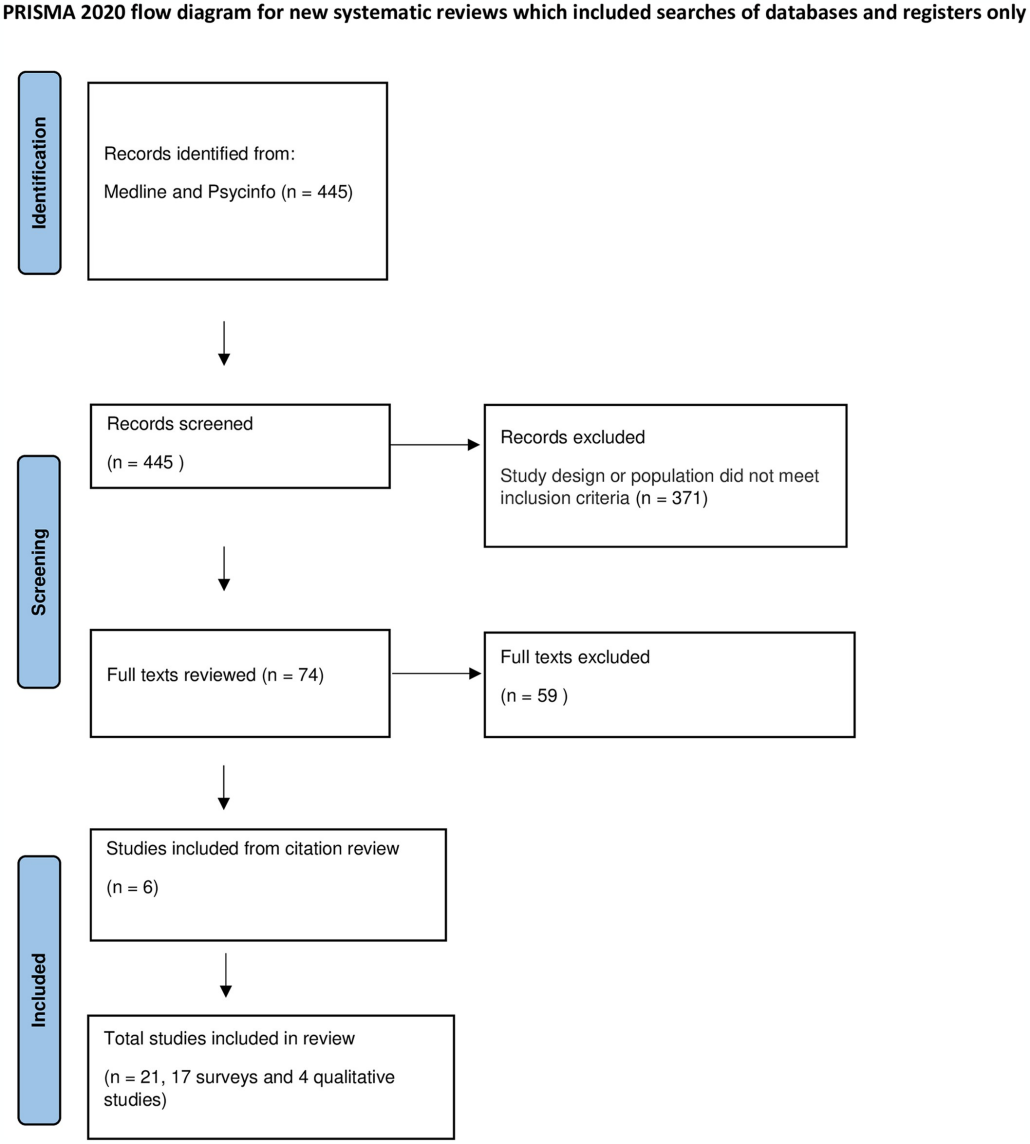
PRISMA flow diagram of paper identification, review, inclusion and exclusion.

An Ovid search was performed using MEDLINE and APA PsycInfo databases using key term combinations of cancer and cirrhosis terms (“liver cirrhosis”, “viral hepatitis”, “hepatitis B”, “hepatitis C”, “liver cancer”), barriers and enabler terms (“surveillance”, “barriers”, “non-adherence”, “limitation”, “refusal”, “patient acceptance of health care”, “patient compliance”, “treatment refusal”, “cultural diversity”) and outcome terms (“hepatocellular carcinoma”, “improve”, “increase uptake”, “adherence”, “improvement”). The dates searched were from January 1, 2012, to August 10, 2023. Articles written in languages other than English were excluded. A PRISMA flow diagram of the search results is shown in Fig 1.

### Extraction

Study characteristics and outcome data were extracted and assessed independently by two reviewers (DM and JEF) using Covidence—Version 2.0 and a standardized form in Microsoft Word. The main components of the assessment were the inclusion and selection of studies, and reporting of study characteristics and outcomes. Data and variables extracted included authors, study years, countries, cohort size, aetiology of liver disease, study methods, study results and quality appraisal.

### Quality appraisal and risk of bias

The Critical Appraisal Skills Programme (CASP) checklist was used by both reviewers to assess the quality of each study and the risk of bias [22]. The studies included have moderate to high risk of bias, and for the purpose of this review, we decided to use a narrative method to report the studies. After removing the high risk of bias studies, there was no discernible difference in the results. Thus, it was decided to report on all these studies for the purpose of this review.

## Results

The search returned 445 unique citations. After screening the titles and abstracts, 371 studies were excluded for not meeting study criteria and 74 studies underwent full text review. Of these, 15 studies met study criteria for inclusion and proceeded to data extraction by both reviewers. A further six studies were found via citation reviews outside of the search strategy, which underwent full text review and proceeded to data extraction. Seventeen of the included studies were descriptive quantitative surveys that included qualitative analyses of unstructured free-text responses to survey questions and four were qualitative studies. Eleven studies were from the USA, three from each of Australia and China and one from each of Taiwan, Thailand and Mongolia and Canada. Study years included 2013–2023. The Aetiology of liver diseases included were chronic liver diseases including hepatitis B and C and cirrhosis. Cohort sizes varied according to the study design and were between 19 to 1000 patients. A flow diagram of the review and exclusion process is shown in Fig 1. Characterestics of the included studies can be found in Tables 1 and 2.

**Table 1:** Patient perspectives.

Author, Year	Country	Aim	Population	Method	Outcomes	Risk of Bias
Chen, 2013	Taiwan	To measure patient perceptions about preventing HCC	400 chronic hepatitis B/ hepatitis C adult patients	Two surveys	Patient health perspectives on preventing HCC related to age, education levels, household income, and knowledge of hepatitis.	Moderate
Li, 2017	USA	To identify patient-level determinants of HCC surveillance awareness and doctor-patient communication regarding liver cancer and compare HCC surveillance awareness with colorectal, prostate, and breast cancer surveillance awareness in patients with chronic liver disease.	467 patients with chronic liver disease from a single tertiary hospital (USA) between 2009–2013	Phone survey	79% reported awareness for the need of liver cancer screening. Patients with higher education, no alcohol usage in the past year, and a cirrhotic liver were more likely to know about liver cancer screening. 50% reported that their doctors had talked to them about liver cancer. Patients with higher education, and those with a cirrhotic liver were more likely to report remembering their doctor talking to them about liver cancer.	High
Farvardin, 2017	USA	To characterize patient level knowledge, attitudes, and barriers regarding HCC surveillance and their association with receipt of HCC surveillance	541 Adult patients with cirrhosis from one urban hospital	Survey	Most patients knew cirrhosis was a risk for HCC and believed HCC surveillance was important and detected early stage cancer 20–30% believed HCC surveillance was unnecessary in the absence of symptoms or could be stopped if scans were normal Nearly half believed eating a healthy diet could lower risk of HCC and not require further HCC surveillance Factors associated with higher levels of overall knowledge were Caucasian, English speaking background, having HCV-related cirrhosis, cirrhosis, being engaged in hepatology subspecialty care, higher educational level and high perceived likelihood of dying from HCC Barriers included difficulty accessing ultrasound appointments, cost and transportation	Moderate
Xu, 2017	China	1. To investigate HCC surveillance practice among high-risk Chinese patients 2. Identify the sociodemographic and clinical factors related to HCC surveillance practice 3. Examine the association of sociodemographic and clinical factors with HCC surveillance knowledge 4. Identify the barriers to HCC surveillance.	352 Patients from outpatient clinics with a high risk of developing HCC (chronic hepatitis B/hepatitis C)	Survey	Patients with better knowledge of viral hepatitis, HCC, and screening guidelines were more likely to be screened. Younger patients had better knowledge. Patients residing in rural regions had less knowledge 55% of patients with routine screening and 63% with irregular screening did not know the purpose of the liver AFP test. “Not aware that screening for HCC exists” and “lack of recommendation from physicians” were the lead reasons given for not undergoing screening	High
Sheppard-Law 2018	Australia	To investigate hepatocellular carcinoma surveillance utilisation and factors associated with utilisation among patients prescribed hepatitis B virus anti-viral therapy and at risk of hepatocellular carcinoma.	177 adult patients prescribed hepatitis B virus anti-viral therapy and at risk of hepatocellular carcinoma	Survey	Patient utilisation of HCC screening programs were significantly associated with patient awareness and patient knowledge of regular six-monthly screening, with ORs of 4.2 and 8.8, respectively. Participants knowledge that screening should be undertaken every six months was associated with a longer duration of therapy (>4 years vs.≤4 years) and pre-treatment education. Participants reporting a family history of HCC were less likely to be aware of HCC surveillance.	High
Allard, 2018	Australia	Explore African- Australians’ understanding of surveillance and the risks associated with chronic hepatitis B.	19 African born Australian adults living with chronic hepatitis B	Semi structured interview	Risk of liver cancer overestimated as ’big’ or up to 50%, particularly if they knew someone who had died from liver cancer Younger patients perceived liver problems linked with ageing Limited knowledge of hepatitis B and confusion with HIV/AIDS Older patients accepted diagnosis by understanding that chronic hepatitis B was common in their family and community Societal stigma and discrimination outside immediate family—exclusion by community members and negative experiences in healthcare, employment and educational settings; and lack of awareness that such behaviour infringed human rights	Moderate
Dai, 2020	China	To devise a scale for HCC surveillance knowledge and attitude among high-risk patients and identify sociodemographic, clinical, and psychological factors influencing each domain of HCC related knowledge.	380 Patients with chronic liver disease at risk of HCC	Survey	Patients with college education level or above and those with a longer duration from diagnosis of chronic liver disease had better HCC knowledge. Patients who fully trusted in doctors had higher HCC knowledge.	Moderate
Singal, 2021	USA	To characterize patient knowledge, attitudes, and barriers of HCC surveillance and their association with surveillance uptake	1020 adult patients with compensated cirrhosis	Telephone survey	50% reported barriers to HCC surveillance, including cost, scheduling, and transportation. 50% expressed worry about ability to pay medical bills and nearly 25% reported delays in medical care including HCC surveillance related to financial distress.	High
Li, 2022	China	To explore barriers to hepatocellular carcinoma surveillance among patients with hepatitis B	23 hepatitis B patients	Face-to-face semi-structured interviews	Lack of concern of hepatitis B threat Feeling ‘protected’ from HCC Lack of awareness of HCC screening Discounting HCC screening to hide the disease Insufficient family and community support due to lack of community awareness of HCC Lack of shared decision-making in the health system Inadequate rural reimbursement policy.	Moderate
Teerasarntipan, 2022	Thailand	To identify HCC surveillance barriers from both physician’s and patient’s perspectives	Patients with high risks for developing HCC who were followed up at the liver clinic	Survey	Financial concerns were not a significant barrier to surveillance Doctors not ordering the ultrasounds for patients was a major barrier to timely surveillance.	Moderate

**Table 2:** Clinician perspectives.

Author, Year	Country	Aim	Population	Method	Outcomes	Risk of Bias
Han, 2014	USA	To identify barriers and facilitators of patients diagnosed with viral hepatitis to attend follow up visit from the perspective of primary care physicians (PCPs).	20 Multi-ethnic Primary Care Physicians	Semi-structured interviews	Barriers included busy patients/busy doctors, miscommunication and language, healthcare costs, cultural beliefs, lack of patients’ knowledge, lack of providers’ knowledge, presence of community-based viral hepatitis surveillance initiatives Facilitators included fear of illness, preference for follow-up with physicians within the community, Community support, engagement of PCPs for specialty care, use of patient’s navigators and insurance status	High
Dalton-Fitzgerald 2015	Australia	To explore provider- and practice-level factors associated with guideline-consistent recommendations for HCC surveillance in patients with cirrhosis.	131 practising primary care providers	Web based survey	>90% believed HCC surveillance is their responsibility; however, they were unclear about how best to perform surveillance 90% of providers believed AFP was an effective surveillance test when used alone Two thirds reported performing annual, instead of biannual surveillance. Most reported believing CT and MRI were effective as HCC surveillance tests 25% reported using CT/MRI intermittently as surveillance tests. Barriers to implementation, including not being up-to-date with current guidelines and having more important issues to manage in the clinic.	High
McGowan, 2015	USA	To assess Primary Care Providers’ knowledge and practice of HCC surveillance	391 Primary Care Providers	Mail Survey	45% of primary care providers (PCPs) who see cirrhosis patients in North Carolina recommend surveillance. Approximately 70% of PCPs screen because they believe evidence supports it. 42% understood some medical associations recommend it. Of the majority not screening, 84% deferred to subspecialists to recommend or consider surveillance, and 24% were unaware of surveillance recommendations 8% did not screen as they felt an uncertain benefit—a large number of PCPs may recommend surveillance, if guidance and education are provided	High
Fitzgerald, 2016	USA	To examine hepatitis B and HCC knowledge, surveillance practices, and barriers to surveillance among PCPs in NYC treating immigrants from high-risk regions	109 Primary Care Physicians	Survey	Providers overwhelmingly correctly identified ultrasound as the screening modality of choice for HCC (92%), but only 64% correctly identified that ultrasound screening should be performed every 6–12 months. “lack of patients’ awareness of hepatitis and liver cancer risk”, and “lack of insurance or cost to the patient” as the main barriers faced by patients. Almost one-third of physicians reported not routinely recommending HCC screening in patients testing positive for hepatitis B. Most common barrier cited by providers for providing either hepatitis B or HCC screening was lack of clear guidelines, and this was similar in both African and Asian populations.	Moderate
Kim, 2016	Mongolia	To evaluate Mongolian physicians’ knowledge of liver disease, their comfort level in the management of liver disease, their access and perceived barriers to surveillance, diagnosis and treatment and their proposed solutions	Physicians from all major provinces of Mongolia attending a continuing medical education liver symposium	Survey	The main perceived barriers to screening were inability to pay for diagnostic tests, lack of clinical guidelines and poor patient awareness with a major HCC screening barrier being cost.	High
Mukhtar, 2017	USA	To describe provider practices, as well as knowledge, attitudes, and barriers to the prevention and management of hepatitis B in a diverse patient population across healthcare systems	277 Primary care providers	Mail Survey	40% were unfamiliar with hepatitis B management guidelines 33% of providers were unaware that hepatitis B induced liver cancer can occur in the absence of cirrhosis	Moderate
Lun Yau, 2019	Canada	To examine specialist surveillance practices, mechanisms, and obstacles to surveillance for HCC among persons living with chronic hepatitis B	71 members of the Canadian Association for the Study of the Liver	Online survey	Significant variability in screening and inconsistency in following published guidelines, even among specialists. 42.9% of respondents reported the lack of an automatic recall system 30.0% reported patient non-compliance 17.1% had limited access to US or MRI.	Moderate
Simmons, 2019	USA	To investigate knowledge and barriers to HCC surveillance among PCPs at university-affiliated academic medical centres.	Primary Care Providers (PCPs)	Anonymous electronic survey	Provider-reported barriers to HCC surveillance included limited time in the clinic, competing clinical concerns, and being out-of-date with surveillance guidelines.	High
Mahfouz, 2020	USA	1. To evaluate knowledge of surveillance strategies for hepatitis B and HCC in trainees 2. Provider perspectives on barriers and facilitators to surveillance for hepatitis B and HCC among trainees from three hospitals in Miami.	183 Trainees from three different institutions in Miami	Paper-based survey	The top three barriers preventing screening for HCC in hepatitis B patients were: lack of clarity of HCC guidelines, 47%; uncertainty/lack of awareness about HCC guidelines, 40%; and patient financial barriers, 29%. Only 17% responded that there were no barriers to HCC screening.	High
Jacobs, 2022	USA	To achieve a greater understanding of the challenges in HCC care	214 Healthcare providers	Mixed methods including a qualitative (semi-structured interviews) component followed by online survey	Challenges included: (1) adherence to established surveillance criteria; (2) use of appropriate imaging for diagnosis; (3) making the decision to perform a diagnostic biopsy.	Moderate
Kim, 2022	USA	To assess: (1) existing HCC surveillance practices, and investigate whether gastroenterology and hepatology providers’ (2) decisions to screen patients for HCC and their choice of surveillance test are influenced by patient-specific HCC risk	Gastroenterology and hepatology providers (40%faculty physicians, 21% advanced practice providers, 39% fellow-trainees) from 26 US medical centres in 17 states	Anonymous web-based survey	Barriers to HCC surveillance included limited clinical time and patients’ difficulties with cost of care, transportation, and scheduling HCC guidelines were an important facilitator of surveillance Trainees were most likely to order ultrasound alone, whereas advanced practitioners were most likely to order cross-sectional imaging and surveillance for patients with annual HCC risks of 0.1% and 0.5%.	High
Teerasarntipan, 2022	Thailand	To identify HCC surveillance barriers from both physician’s and patient’s perspectives	Physicians working at different hospital levels (community, secondary, and tertiary centres) located in all geographic regions of Thailand (57 of 77 provinces).	Survey	More than half of physicians failed to suggest proper surveillance to patients at risk for HCC Insufficient knowledge regarding indication, tool, and interval for HCC surveillance. Most physicians believed that the surveillance was a responsibility shared by all healthcare providers regardless of medical specialty and hospital limitations. Agreed that surveillance was cost-effective and did not increase their workload. A minority of physicians responded that they had been influenced by patients’ history of heavy alcohol and were less likely to offer HCC surveillance for alcoholic patients. Almost half of physicians reported having limited access to US machines, especially in community hospitals. The AFP test was unavailable in 72% of community hospitals. Due to resource limitation, surveillance might still be difficult to achieve despite sufficient physicians’ knowledge and proactive attitudes toward surveillance Financial concerns were not a significant barrier to surveillance from both patients’ and physicians’ perspective, indicating that patients’	Moderate

## Analysis

Analysis was performed by the principal reviewer. A thematic synthesis using a modified version of Bronfenbrenner’s SEM (1979) was used to analyse data from included studies, displaying the barriers to HCC surveillance from both the patient and physician perspectives [23]. The 21 identified studies underwent thematic synthesis with four themes identified and categorised:

- Knowledge of liver cancer and HCC surveillance;
- traditional health beliefs, culture, stigma and discrimination impacts on HCC surveillance uptake;
- logistical barriers, and
- unclear HCC surveillance guidelines.

**Fig 2:**
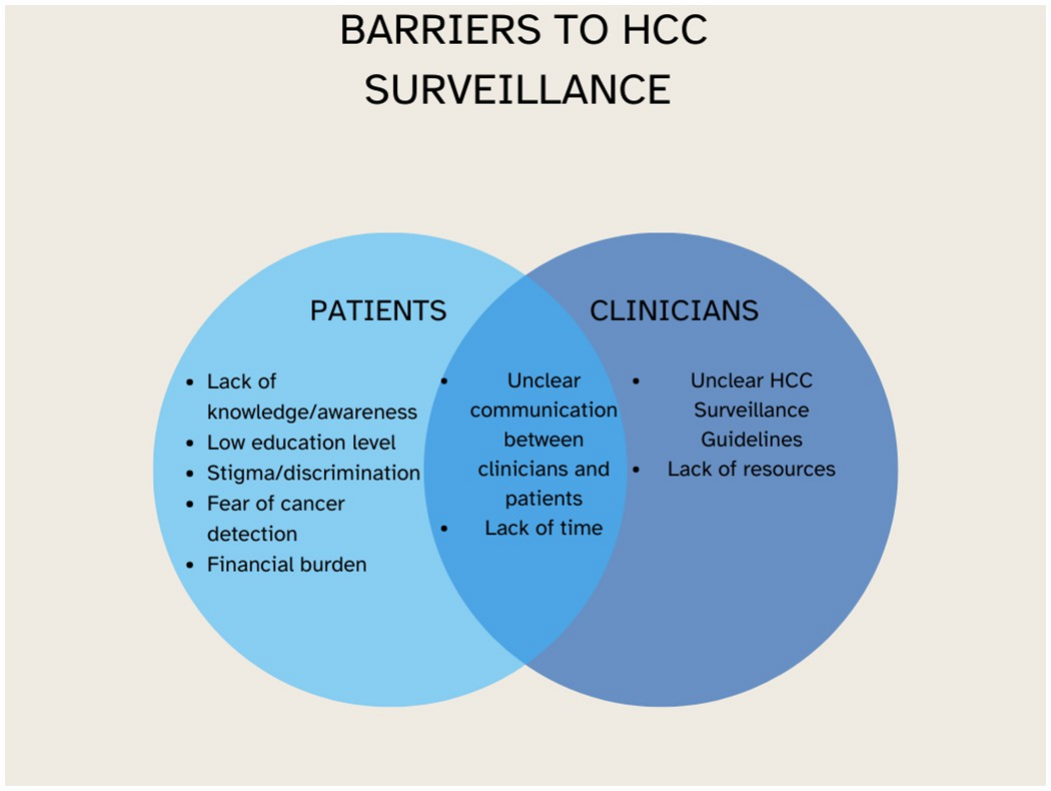
Summary of barriers to HCC Surveillance from both patients and clinicians’ perspectives.

A summary of the barriers to HCC surveillance identified by patients and clinicians as a result of the thematic analysis is shown in Fig 2.

## Patient perspectives

Twelve papers explored barriers to HCC surveillance from the patients’ perspective (Table 1). The themes identified from the analysis were knowledge of liver cancer and HCC surveillance, Traditional health beliefs, culture, stigma and discrimination impacts on HCC surveillance uptake and Logistical barriers.

### Knowledge of liver cancer and HCC surveillance

Several studies from Asia-Pacific region countries with well-established HCC surveillance programs have explored the impact of knowledge and awareness on HCC surveillance uptake. A survey study by Chen and colleagues conducted in a single medical institution in Taiwan recruited 400 patients living with chronic hepatitis B and/or hepatitis C and explored patients’ perspectives on HCC prevention [24]. Responses from the survey indicated that patient knowledge about HCC prevention was closely associated with age, education level, household income and knowledge of viral hepatitis. The study recommended detailed communication and education from healthcare providers regarding viral hepatitis related HCC as essential to prevent or detect HCC early, especially in elderly individuals and people from low socioeconomic backgrounds and education levels who had lower HCC knowledge.

Two studies from China reported knowledge barriers to HCC surveillance [25, 26]. A survey of 352 patients with chronic hepatitis B and/or hepatitis C from hospital outpatient clinics investigated HCC surveillance practices and the association of sociodemographic factors with surveillance knowledge and uptake [25]. Factors associated with better knowledge and likelihood of HCC surveillance uptake included greater knowledge of viral hepatitis and cancer surveillance guidelines, with patients with high school or college younger patients having higher levels of knowledge being more likely to undergo routine screening. Household income and insurance status did not impact surveillance knowledge or uptake by patients, however participants living in rural provinces reported less knowledge of hepatitis B and liver cancer than those living in urban provinces. The study demonstrated a deficiency in HCC surveillance awareness among patients, with many undergoing AFP surveillance simply because they were asked to do so by their doctors without understanding why. While the study noted that physicians in China are often regarded as authoritative figures, and it is essential for clinicians to specifically recommend HCC surveillance to patients to increase uptake, many participants stated that this recommendation to undergo HCC surveillance has not been made by their physicians.

Furthermore, Dai et al. conducted a study involving 380 patients with chronic liver disease in China, aiming to identify sociodemographic, clinical, and psychological factors influencing HCC-related knowledge [26]. Responses to this survey showed that patients with a college education or above and those with a longer duration from diagnosis of chronic liver disease had better knowledge of HCC including surveillance, possibly due to more frequent clinical visits, thereby increasing opportunities for patients to be exposed to HCC education.

In Australia, Sheppard-Law et al. surveyed 177 patients living with hepatitis B across three tertiary hospital clinics, aiming to investigate the factors associated with HCC surveillance [27]. Participants’ country of birth according to WHO region was Western Pacific (78%), Africa (8.4%), Europe (5.1%), and South East Asia (8.5%). Patients in this study were more likely to uptake HCC surveillance if they had higher levels of knowledge and awareness of the importance of six-monthly surveillance, which was associated with pre-hepatitis B treatment education, longer duration of therapy, and attendance at a hepatology specialist clinic. Interestingly, participants who reported a family history of HCC were less likely to be aware that six monthly screening was recommended.

Another Australian study by Allard et al explored African-born Australians’ understanding of HCC surveillance and the risks associated with chronic hepatitis B using semi-structured interviews conducted with 19 patients recruited from a hospital and three recruited from community clinics [28]. Participants mostly understood the significance of the impact of hepatitis B on the liver and reported that the risk of liver cancer was high, particularly among people who had known someone who had died from this cancer. Moreover, younger patients cited liver problems as “associated with aging” and the relationship between regular HCC surveillance to reduce the risk of liver cancer death was generally not understood, highlighting that lack of knowledge were major barriers to HCC surveillance uptake in this key population.

Several studies have been conducted in the USA exploring factors impacting HCC surveillance uptake. Li et al. conducted a phone survey with 467 patients with chronic liver disease attending a single tertiary hospital liver clinic in St Louis, USA. This study aimed to identify barriers and enablers of HCC screening. The study compared HCC screening awareness with colorectal, prostate, and breast cancer screening awareness in patients with chronic liver disease. Of patients with liver disease, 79% reported that they were aware of liver cancer screening. Patients with higher education, no alcohol use in the past year, and a cirrhotic liver were more likely to know about liver cancer screening. Fifty percent of patients reported that their doctors had talked to them about liver cancer, with the prevalence of doctor-patient communication not differing by sex, age, BMI, smoking status, alcohol usage, or frequency of doctor visits. However, Caucasians, patients with higher education levels, and those with cirrhotic livers were more likely to remember their doctor talking to them about liver cancer [29]. Also in the USA, Farvardin et al. conducted a study of 541 patients living with cirrhosis, aiming to identify patient-level knowledge and barriers to HCC surveillance uptake and their association with receipt of HCC surveillance in an ethnically diverse and socioeconomically disadvantaged cohort of 541 participants from Dallas County [30]. Factors associated with higher levels of overall knowledge included being Caucasian, English as a primary language, hepatitis C-related cirrhosis, receiving specialist care, higher educational attainment, and high perceived likelihood of dying from HCC, while factors associated with lower levels of knowledge included living alone, and lack of insurance.

### Traditional health beliefs, culture, stigma and discrimination impacts on HCC surveillance uptake

Traditional health beliefs and stigma can impact HCC surveillance uptake and have been directly explored in some studies. For example, discrimination against people with hepatitis B, health beliefs and culture in China are identified as barriers to HCC surveillance uptake, with stigma linked in part to myths surrounding how hepatitis B is transmitted and reducing the willingness of people with hepatitis B to disclose their infection status, access health services for their infection, or undergo HCC surveillance [25, 26, 31]. A survey study conducted by Dai et al. showed that patients with a greater trust in doctors had greater levels of knowledge of HCC risks and surveillance [31]. Main themes in barriers to surveillance reported by 23 hepatitis B patients included their capacity to endure physical suffering, family priorities and responsibilities, and the lack of support from the community, health systems and policies.

In the Australian study by Allard et al, diagnosis of HCC was described as a major life event with significant psychosocial ramifications [28]. Due to limited knowledge of chronic hepatitis B among the African community in Melbourne, confusion with HIV/AIDS, and related stigma experienced within the community, a diagnosis of HCC or uptake of HCC surveillance was perceived as potentially leading to community exclusion and negative experiences in healthcare, employment, and educational settings. Most participants were unaware that social exclusion related to blood borne infections infringed human rights and were unaware that disclosure of viral status was only mandatory in limited situations. Some older patients accepted the diagnosis by understanding that hepatitis B was common in their family and community, while younger patients were more likely to self-stigmatize when they did not know others living with chronic liver disease. Additionally, when it came to intimate relationships, it was reported that disclosure was sometimes associated with the risk of being questioned about fidelity and its related impact on their relationships, in part due to the risk for HCC being related to hepatitis B as being understood as a sexually transmitted infection. While these studies have limited samples from specific communities that cannot be generalised more broadly, they highlight the impact stigma and community perceptions can have on HCC surveillance uptake among at-risk populations.

### Logistical barriers

Logistical and health system barriers to HCC surveillance uptake differ significantly between countries, however they remain commonly cited barriers to HCC surveillance uptake by patients and clinicians in both low and high resource settings.

Two studies from the United States highlighted significant logistical barriers to HCC surveillance, delivery, and uptake. Farvardin et al. highlighted the main barriers to HCC surveillance uptake experienced by patients being difficulty in scheduling liver ultrasound appointments, out-of-pocket cost to patients of HCC surveillance tests, uncertainty of where to access HCC surveillance, and difficulties with transportation. At least one of these barriers was reported by half of respondents, while 23% reported several barriers. Less commonly reported barriers included fear of poor surveillance test efficacy, fear of finding cancer, the time commitment required to attend surveillance, and fear of pain from surveillance tests [30].

Singal et al. conducted a telephone survey of 1020 patients living with cirrhosis within three different health systems in the USA, to characterize patient knowledge, attitudes, and barriers to HCC surveillance and their association with surveillance uptake in a racially and socioeconomically diverse cohort of patients. Similarly, nearly half of the patients in this study reported logistical barriers to HCC surveillance, including cost, scheduling, and transportation [32].

Similar logistical barriers to HCC surveillance have been reported in Asian countries. In China, Xu et al. surveyed 352 patients with chronic hepatitis B and/or hepatitis C from hospital outpatient clinics. They found patient-perceived logistical barriers to HCC surveillance included lack of time, difficulty accessing medical facilities and fear of cancer detection [25]. A survey study from Thailand where the universal medical coverage scheme supports HCC surveillance to all patients with high-risk liver disease, found that financial concerns were not a significant barrier to surveillance uptake. However, the lack of ultrasounds being requested by physicians was associated with a low rate of HCC surveillance uptake, reflecting health systems barriers in busy hospital settings [33].

## Clinician perspectives

Twelve papers explored the clinical and health system aspects that influence surveillance uptake as perceived by health workers (Table 2), including perspectives from nurses, specialists and primary care physicians/ general practitioners. The themes identified from the analysis were knowledge of liver cancer and HCC surveillance, Inconsistent guidelines and logistical barriers.

### Knowledge of liver cancer and HCC surveillance

Studies among primary care physicians in the USA described lack of knowledge being the key barrier to implementing surveillance for their patients. In a study in New York, Han et al. examined barriers and enablers to care for patients with viral hepatitis from the perspective of ethnically diverse primary care physicians through semi-structured interviews. The primary care physicians self-identified as being of Korean, Chinese, Egyptian, or Russian ethnicity, fluent in their native language, with 65% of their patients being of the same cultural and language background. Some reported barriers to care included busy patients and doctors, miscommunication and language, cultural beliefs, healthcare costs, lack of patient and provider knowledge, and lack of community-based viral hepatitis surveillance initiatives to identify those at potential risk for HCC. Alternatively, some of the facilitators of care identified included fear of illness, preference for follow-up with physicians within the community, community support, engagement of primary care physicians for specialty care, and insurance status [34]. Furthermore, McGowan et al aimed to understand North Carolina primary care providers’ knowledge and practice of HCC surveillance via a survey with the results showing only 45% who see cirrhosis patients recommend surveillance and of those, only 70% did so because they believe evidence supports it. Of the primary care providers who did not conduct HCC surveillance, 84% referred to specialists to provide surveillance and 24% were unaware of surveillance recommendations. Only 8% did not provide HCC surveillance due to lack of belief in a survival benefit [35].

### Inconsistent guidelines

A survey of 109 primary care physicians in New York treating immigrants from countries with endemic viral hepatitis described several HCC practices and barriers [36]. HCC surveillance for hepatitis B patients was recommended by just over two-thirds of the 93 providers responding to that question. Of 85 respondents, 78% determined that HCC surveillance should be performed for immigrant patients from high-risk regions living with hepatitis B, even if no symptoms are present while over half of the providers stated that there was a lack of clear HCC surveillance guidelines, while a further proportion expressed lack of clarity in existing HCC surveillance guidelines for which groups should receive surveillance. Even though most providers identified that ultrasound should be used for surveillance, only 64% stated that it should be performed every 6 to 12 months. Moreover, a web-based survey from Australia, explored provider- and practice-level factors associated with guideline-consistent recommendations for HCC surveillance in patients with cirrhosis among 131 primary care physicians. Barriers to surveillance included misconceptions about how best to perform surveillance, perceived and experienced difficulties in effectively implementing surveillance, not being up-to-date with current HCC surveillance guidelines, and not receiving systems-level reminders, such as computer-based prompts for HCC surveillance. In contrast to guideline recommendations for 6 monthly liver ultrasounds, 90% of respondents interviewed believed alpha fetoprotein (AFP) was an effective surveillance test when used alone, with two-thirds reporting performing annual instead of biannual HCC surveillance. Most respondents believed that computerized tomography (CT) and Magnetic Resonance Imaging (MRI) were effective as HCC surveillance tests, and one-fourth of the providers reported using CT/MRI intermittently as surveillance tests in their patients instead of liver ultrasound. Thus, several HCC surveillance recommendations and practices reported by primary care physicians are inconsistent with current guidelines [37].

In an additional study from the USA, Mukhtar et al. aimed to describe primary care physicians’ knowledge of and barriers to the management of hepatitis B in a culturally diverse patient cohort across several healthcare systems in San Francisco. This was done via a mail survey with 277 primary care physicians participating (41.3% response rate). Major barriers included unfamiliarity with and lack of clarity of hepatitis B management guidelines and HCC surveillance, and a lack of awareness of when hepatitis B should be treated or understanding that hepatitis B-induced liver cancer can occur in the absence of cirrhosis. Furthermore, although most providers performed HCC surveillance, only half performed HCC surveillance in >75% of hepatitis B patients [38]. Simmons et al. explored the knowledge and barriers to HCC surveillance among primary care physicians at university-affiliated academic medical centres in the USA via an electronic survey. Barriers to HCC surveillance in the study included limited time in the clinic, competing clinical concerns, and not being aware of current surveillance guidelines [39]. Barriers to HCC surveillance were evident among medical graduates. Mahfouz et al. surveyed medical trainees from three different institutions in Miami to evaluate the knowledge of perspectives on barriers and facilitators for surveillance for hepatitis B and HCC. Barriers cited by participants included lack of clarity in HCC guidelines (46.7%), lack of awareness about HCC guidelines (39.6%), and patient financial barriers (28.6%), with only 16.5% responding that there were no barriers to HCC surveillance. On the other hand, some respondents were highly likely to screen for HCC if evidence strongly suggested that surveillance leads to decreased mortality (95% agree/strongly agree) or if surveillance was recommended by a national organization (87% agree/strongly agree) [40]. This study highlights the importance of adequate education for medical trainees to facilitate HCC surveillance delivery once they independently practice medicine.

Specialists expressed similar issues to primary care providers, in that unclear and inconsistent guidelines reduced HCC surveillance uptake. Kim et al. surveyed 654 gastroenterology and hepatology providers from 26 medical centres in 17 U.S. states, of whom 305 completed the survey (47% response rate). The survey sought to assess existing HCC surveillance practices and investigate whether decisions to screen patients for HCC and their choice of surveillance test were influenced by patient-specific HCC risk. Barriers to surveillance include limited clinical time and patients’ costs of care, transportation, and scheduling. There were significant differences in HCC surveillance recommendations by healthcare provider type: trainees were more likely to order ultrasound alone, whereas experienced providers were more likely to order cross-sectional imaging and surveillance for patients with annual HCC risks of 0.1% and 0.5%. The reasons for these findings may include incomplete awareness of guidelines, trainee modelling after the practices of their faculty physicians, and experienced providers being more concerned about missing HCC and related legal ramifications in the American health system, prompting more aggressive surveillance, while the availability of HCC-related guidelines was an important facilitator of surveillance [41]. Another mixed methods study, including a qualitative (semi-structured interviews) component followed by online survey among 214 healthcare providers in the USA aimed to understand HCC care challenges. Healthcare providers included oncologists, hepatologists, oncology physician assistants, oncology nurse practitioners, and interventional radiologists that were involved in HCC care. The main barriers to HCC surveillance that were reported included adherence to established surveillance criteria, use of appropriate imaging for diagnosis and making the decision to perform a diagnostic biopsy [42].

In Canada, an online survey among 71 members of the Canadian Association for the Study of the Liver, most being gastroenterology and hepatology providers, was conducted to understand their HCC surveillance practices and barriers among patients living with chronic hepatitis B [43]. The survey results showed significant differences and inconsistency in surveillance practices and in following guidelines. The main barriers reported included patient non-compliance, a lack of an automated recall system and limited access to ultrasound machine or MRIs.

### Logistical barriers

In the Asia Pacific region, the main barrier to HCC surveillance perceived among physicians in Mongolia included cost and the inability of patients to pay for diagnostic tests, with other barriers cited included the of lack of clinical guidelines and poor patient awareness [44]. A survey conducted among physicians in Thailand showed that more than half failed to suggest proper surveillance to patients at risk for HCC, despite most believing that HCC surveillance was a shared responsibility by all healthcare providers and that it was cost-effective. The survey results highlighted that some of the barriers to screening was a lack of knowledge regarding intervals of HCC surveillance, limited access to ultrasound machines especially in rural areas and that the AFP test was unavailable in over 70% of community hospitals [33].

## Discussion

This is the first systematic review that has thematically analysed qualitative data describing the perceived and experienced barriers to and enablers of HCC surveillance uptake from both patient and clinician perspectives to inform the development of further clinical and health system interventions. In conducting a thematic synthesis, this review addresses a gap in knowledge regarding the identification of cross-cultural factors influencing HCC surveillance knowledge and uptake. This data analysis provides a broader insight into the drivers of health-related attitudes and behaviours, including identifying the intersectionality between culture, sociodemographic factors, personal experience, and health service access and utilisation. These data highlight common barriers and enablers experienced across a wide range of contexts to inform interventions to improve HCC uptake, but also highlight context-specific differences that may guide more tailored approaches to health promotion and health system interventions to improve HCC outcomes.

Findings from this thematic synthesis identified that lack of knowledge, logistical barriers, stigma and discrimination and unclear HCC surveillance guidelines are the key barriers to uptake of HCC surveillance.

Among patients, logistical barriers, including out-of-pocket healthcare and transportation costs, and a lack of awareness and knowledge were the most frequently cited barriers. Results of studies exploring patient perspectives reinforce that barriers are experienced by patients across different health settings, cultures, and regions and highlight the critical need for changes in the health system to improve engagement in HCC surveillance. In many cultures, patients rely on and trust their physicians when it comes to any health issues, and if physicians do not specifically recommend surveillance, patients may not seek further advice elsewhere. Hence, they will fail to be screened and may only present back at medical care when they have already developed HCC. This might have implications in some cultures as to who should provide HCC surveillance and whether it should be the GP, specialist or nurse practitioner.

Clinicians have highlighted the need for provider education and system-level interventions to optimize HCC surveillance effectiveness in clinical practice. The consistency and clarity of HCC surveillance guidelines is essential, particularly to ensure that current guidelines are accessible and visible to primary care physicians who may not see patients with liver disease frequently in their clinics. Health system-level interventions, such as electronic health reminders and mailed outreach interventions, significantly increase HCC surveillance rates compared to opportunistic visit-based surveillance. Furthermore, improving the human rights of people with hepatitis B by improving health service access through stigma and discrimination reduction and community awareness and education are critical to facilitating increased HCC surveillance uptake by both patients and providers. There are many barriers, each requiring solutions at the patient and provider levels, with further research needed to identify potential interventions.

This review has several limitations. First, studies published since 2012 and in English were included, with the possibility that studies with significant data pertinent to our systematic review from outside the study period may have been missed. We selected our study period to reflect published international HCC guidelines that would be expected to impact surveillance uptake after 2012 [9]. Second, the studies in this review represent several regions globally; however, and no studies were available from a European perspective within the study timeframe. Further data from culturally and geographically diverse populations are needed to understand additional barriers and enablers to HCC surveillance uptake, particularly to understand the role of culture, society and traditional understandings of medicine and their intersectionality with marginalisation and stigma on HCC surveillance knowledge and uptake. Third, most of the studies explored more barriers than enablers of HCC surveillance. Fourth, most studies had a limited setting, such as taking place in one health service, therefore the results could not be generalised to HCC surveillance populations outside the health settings described. Fifth, studies were self-reported, and thus may have been affected by recall and information bias. Finally, the heterogeneous methodologies of included studies limited meaningful synthesis of results. We acknowledge that surveys have a risk of bias such as participation bias and recall bias. However, this allowed the exploration of barriers and enablers among larger and potentially more representative populations which triangulates the findings of this study.

### Conclusions

Our systematic review of qualitative studies highlighted several key barriers to HCC surveillance uptake as perceived by both patients and clinicians, including lack of knowledge, conflicting guidelines and logistical barriers. Further qualitative studies representing greater diversity among participants from different health system and resource contexts are needed to better inform design of effective HCC surveillance programs and health promotion strategies.

## Supporting information

S1 Data CASP appraisal and risk of bias. (XLSX)

S2 Data List of studies. (XLSX)
